# Effects of Leptin and Body Weight on Inflammation and Knee Osteoarthritis Phenotypes in Female Rats

**DOI:** 10.1002/jbm4.10754

**Published:** 2023-05-05

**Authors:** Yao Fu, Albert Batushansky, Michael Kinter, Janet L. Huebner, Virginia B. Kraus, Timothy M. Griffin

**Affiliations:** ^1^ Aging and Metabolism Research Program Oklahoma Medical Research Foundation Oklahoma City Oklahoma USA; ^2^ Oklahoma Center for Geroscience University of Oklahoma Health Sciences Center Oklahoma City Oklahoma USA; ^3^ Duke Molecular Physiology Institute Duke University, School of Medicine, Duke University Durham North Carolina USA; ^4^ Division of Rheumatology, Department of Medicine Duke University, School of Medicine, Duke University Durham North Carolina USA; ^5^ Department of Biochemistry and Molecular Biology University of Oklahoma Health Sciences Center Oklahoma City Oklahoma USA; ^6^ Veterans Affairs Medical Center Oklahoma City Oklahoma USA

**Keywords:** ADIPOKINES, KNEE, METABOLIC SYNDROME, METABOLISM, OBESITY, OSTEOARTHRITIS

## Abstract

Leptin is a proinflammatory adipokine that contributes to obesity‐associated osteoarthritis (OA), especially in women. However, the extent to which leptin causes knee OA separate from the effect of increased body weight is not clear. We hypothesized that leptin is necessary to induce knee OA in obese female rats but not sufficient to induce knee OA in lean rats lacking systemic metabolic inflammation. The effect of obesity without leptin signaling was modeled by comparing female lean Zucker rats to pair fed obese Zucker rats, which possess mutant *fa* alleles of the leptin receptor gene. The effect of leptin without obesity was modeled in female F344BN F1 hybrid rats by systemically administering recombinant rat leptin versus saline for 23 weeks via osmotic pumps. Primary OA outcomes included cartilage histopathology and subchondral bone micro‐computed tomography. Secondary outcomes included targeted cartilage proteomics, serum inflammation, and synovial fluid inflammation following an acute intra‐articular challenge with interleukin‐1β (IL‐1β). Compared to lean Zucker rats, obese Zucker rats developed more severe tibial osteophytes and focal cartilage lesions in the medial tibial plateau, with modest changes in proximal tibial epiphysis trabecular bone structure. In contrast, exogenous leptin treatment, which increased plasma leptin sixfold without altering body weight, caused mild generalized cartilage fibrillation and reduced Safranin O staining compared to vehicle‐treated animals. Leptin also significantly increased subchondral and trabecular bone volume and bone mineral density in the proximal tibia. Cartilage metabolic and antioxidant enzyme protein levels were substantially elevated with leptin deficiency and minimally suppressed with leptin treatment. In contrast, leptin treatment induced greater changes in systemic and local inflammatory mediators compared to leptin receptor deficiency, including reduced serum IL‐6 and increased synovial fluid IL‐1β. In conclusion, rat models that separately elevate leptin or body weight develop distinct OA‐associated phenotypes, revealing how obesity increases OA pathology through both leptin‐dependent and independent pathways. © 2023 The Authors. *JBMR Plus* published by Wiley Periodicals LLC on behalf of American Society for Bone and Mineral Research.

## Graphical abstract

Leptin is a proinflammatory adipokine that contributes to obesity‐associated osteoarthritis (OA), especially in women. However, the extent to which leptin causes knee OA separate from the effects of increased body weight is not clear. Rat models that separately elevate leptin or body weight develop distinct OA‐associated phenotypes, revealing how obesity increases OA pathology through leptin dependent and independent pathways.

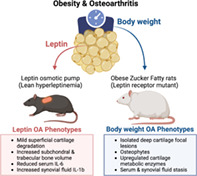

## Introduction

Obesity is a primary risk factor for obesity‐associated osteoarthritis (OA) in both men and women.^(^ ^1^, ^2^ ^)^ The independent roles of obesity‐associated biomechanical and systemic mediators in OA progression are not well understood because both factors covary with obesity.^(^ ^3^ ^)^ Increased body weight with obesity alters knee joint biomechanics, and obesity‐associated changes in limb alignment and knee joint stress have been associated with an increase in the onset and progression of knee OA.^(^ ^4^, ^5^, ^6^, ^7^, ^8^ ^)^ However, obesity also increases hand OA, suggesting that obesity‐associated systemic factors contribute to OA risk.^(^ ^9^ ^)^ Previous studies identified several systemic factors linking obesity to OA,^(^ ^10^ ^)^ including direct and intergenerational effects of diets high in saturated fat,^(^ ^11^, ^12^, ^13^ ^)^ an association with increased intestinal permeability resulting in elevated serum lipopolysaccharide (LPS),^(^ ^14^ ^)^ and the direct contribution of adipose tissue‐derived secreted factors.^(^ ^15^ ^)^ One adipose factor of particular interest to OA is the adipokine leptin, which may explain as much as 50% of the obesity‐associated risk of knee OA in women.^(^ ^16^ ^)^

Leptin is a hormone‐like polypeptide encoded by the obese gene. It is primarily secreted by adipocytes and contributes to the regulation of multiple body systems, including body mass, skeletal development, and immune function.^(^ ^17^ ^)^ Obesity in OA patients is positively associated with leptin gene expression in cartilage and protein abundance in synovial fluid.^(^ ^18^, ^19^ ^)^ In a longitudinal study, elevated serum leptin levels at baseline were associated with a greater risk of knee OA progression in women.^(^ ^20^ ^)^ At the cellular level, leptin functions in a synergistic way with other proinflammatory factors such as IL‐1β to increase nitric oxide production in chondrocytes.^(^ ^21^ ^)^ Leptin also induces matrix metalloproteinase (MMP) expression in chondrocytes via NF‐𝜅B, MAPK, and PI3K/Akt inflammatory pathways.^(^ ^22^, ^23^ ^)^ Studies of cartilage and synovial fluid samples obtained from OA patients undergoing joint replacement surgery showed that the intracellular protein suppressor of cytokine signaling‐3 (SOCS‐3) negatively regulated multiple proinflammatory and procatabolic effects of leptin and was downregulated with obesity.^(^ ^24^, ^25^ ^)^ Although recent studies described additional mechanisms by which leptin promoted cartilage catabolism,^(^ ^26^ ^)^ it is not clear how leptin mediates this risk in vivo because leptin regulates the function of multiple systems, tissues, and cells throughout the body.^(^ ^27^, ^28^ ^)^

An important in vivo finding linking leptin to OA pathology was our observation that mice with impaired leptin signaling (i.e., *ob/ob* and *db/db* mice) were protected from developing knee OA despite becoming extremely obese compared to OA‐susceptible, high‐fat‐diet‐induced obese mice.^(^ ^29^ ^)^ The synergistic effect of leptin with other inflammatory cytokines raises an essential question of whether leptin alone is sufficient to induce OA. We hypothesized that leptin was necessary to induce OA in obese rats, but it was not sufficient by itself to induce OA in lean rats lacking systemic inflammation associated with obesity. Like our prior study of leptin‐mutant obese mice, here we examined obesity in the absence of leptin signaling by comparing female lean Zucker rat control animals to obese Zucker rats, which possess two *fa* mutant alleles of the leptin receptor gene. To study the effect of increasing leptin without increasing body weight, we treated adult F344 BN F1 female hybrid rats with recombinant rat leptin systemically for 23 weeks via an osmotic pump, with control rats receiving saline vehicle solution. Primary OA outcomes included cartilage and synovial histopathology and subchondral bone micro‐CT (μCT). Secondary outcomes included targeted cartilage proteomics, cartilage leptin receptor immunohistochemistry, serum inflammation, and synovial fluid inflammation following an acute intra‐articular challenge with interleukin‐1β (IL‐1β).

## Materials and Methods

### Study design

Experiments were conducted on female rats up to 12 months of age. By 12 months of age, the incidence of death in ad libitum fed female obese Zucker rats is ~20%–30% due to end‐stage renal disease.^(^ ^30^ ^)^ Pair‐feeding obese Zucker rats based on the amount of ad libitum food consumed by lean littermate animals reduces the risk of end‐stage renal disease at 12 months of age with minimal effect on the development of obesity.^(^ ^30^ ^)^ Therefore, obese Zucker rats were pair‐fed as described below to minimize animal loss and control for food consumption. Peripheral leptin infusion dosing was based on results from a prior 7‐day dose–response study (0.03–0.5 mg/day) involving 3‐ and 30‐month‐old male F344BN F1 hybrid rats.^(^ ^31^ ^)^ We selected a dose expected to increase serum leptin twofold with minimal effect on food consumption and body weight to minimize potential confounding factors. Our study focused on young adult female animals for several reasons. First, the association between leptin and OA has primarily been observed in women.^(^ ^16^, ^20^, ^32^, ^33^, ^34^ ^)^ Second, obesity has a greater effect on the magnitude of OA risk in women than men.^(^ ^35^ ^)^ And third, the elevated incidence of knee OA with obesity is most apparent during early and middle adulthood.^(^ ^36^, ^37^ ^)^

### Animals

All procedures were performed in accordance with a protocol approved by the Oklahoma Medical Research Foundation (OMRF) Institutional Animal Care and Use Committee. All animals were single housed in a specific pathogen‐free facility under a controlled environment (22 ± 3°C on 12:12 hour light/dark cycles) in passive ventilated cages with ad libitum access to sterilized water (chlorination between 0.8 and 1.6 ppm). Female obese (*n* = 8) and lean (*n* = 8) Zucker rats were purchased at 4 weeks of age (Harlan Laboratories Inc., Indianapolis, IN, USA). Between 6 and 8 weeks of age, Zucker rats were transitioned from standard chow (~9% to 13% kcal fat) to a defined 10% kcal fat diet (D12450Bi, Research Diets Inc., New Brunswick, NJ, USA). Between 9 and 11 weeks of age, obese Zucker rats were transitioned to pair‐feeding based on the average daily ad libitum food consumption rate of lean Zucker rats from the prior age‐matched week. Food was provided daily to the obese Zucker rats between 09:00 and 11:00 AM. The amount of food varied from 11 to 15 g/day and was continued until the animals were euthanized at 42 weeks of age. Exogenous leptin treatment studies were conducted on female F344BN F1 hybrid rats purchased from the National Institute of Aging (NIA) Aging Rodent Colony at 24 weeks of age. Following the start of leptin (*n* = 8) or saline (*n* = 8) treatment (random, nonblinded allocation), animals were transitioned to a defined 10% fat diet (D12450Bi, Research Diets), provided ad libitum until the animals were euthanized at 54 weeks of age. A second set of animals was purchased to test the effect of an acute intra‐articular cytokine challenge on joint inflammation, as described in detail below. For this experiment, female obese (*n* = 12) and lean (*n* = 12) Zucker rats were purchased from Harlan at 9 weeks of age, and female F344BN F1 hybrid rats (*n* = 20) were purchased from the NIA Aging Rodent Colony at 11 months of age. Animals were fed NIH31 chow diet ad libitum for these experiments. Zucker rats were euthanized at 10 weeks of age, and F344BN F1 hybrid rats were euthanized at 12 months of age.

### Leptin infusion and plasma monitoring

At 31 weeks of age, F344BN F1 rats were anesthetized by isoflurane inhalation to implant mini‐osmotic pumps (Model 2006, ALZET Osmotic Pumps, Cupertino, CA, USA) into a subcutaneous pocket on the dorsal surface adjacent to the scapula. Pumps were loaded with either 1 g/mL recombinant rat leptin (598‐LP, R&D Systems, Minneapolis, MN, USA) in 5 mM Tris–HCl or saline in 5 mM Tris–HCl following the manufacturer's instructions. Based on a diffusion rate of 0.15 μL/h, rats received 3.6 μg leptin per day. Pumps were serially replaced four times over a 23‐week period at 35‐ to 42‐day intervals. For the acute intra‐articular cytokine challenge experiment, a single pump was inserted when animals were 11 months old. Blood was collected during osmotic pump replacement via the lateral saphenous vein and placed in EDTA‐coated tubes. Plasma was obtained by centrifugation for 20 minutes at 2000 g within 30 minutes of collection. Plasma leptin concentrations were measured by immunoassay (MOB00, R&D Systems) following the manufacturer's instructions. The average interassay coefficient of variation (CV) (%) was 5.9%. If a sample was below the lowest level of detection (LLOD), a value of half the LLOD (15.9 pg/mL) was imputed for the purpose of statistical analysis.

### Adipose tissue quantification by MRI

To evaluate the effects of leptin infusion on adiposity, we quantified the fraction of adipose to nonadipose tissue volume by MRI, as previously described.^(^ ^38^ ^)^ Briefly, after 16 weeks of leptin infusion, 47‐week‐old F344BN F1 rats were anesthetized with isoflurane and scanned on a 7‐Tesla, 30‐cm horizontal bore USR Bruker system equipped with an AVANCE I console. A quadrature coil (150 mm ID, 266 mm length) was matched and tuned to 300 MHz for pulse transmission and signal detection. A RARE “water‐suppressed” image sequence was acquired for adipose tissue quantification (1300 ms repetition time, 15 ms echo time, 25 contiguous horizontal slices of 3 mm thickness with 150 × 80 mm field of view and 384 × 256 image matrix). A Mathematica (version 6.0; Wolfram Research, Champaign, IL, USA) notebook was developed to calculate the relative adipose tissue volume by automated segmentation procedures of user‐defined anatomic regions.

### Serum cytokines and biomarkers

Serum was collected when rats were euthanized. Serum concentrations of interleukin‐6 (IL‐6) and hyaluronic acid (HA) were quantified by rat‐specific sandwich enzyme‐linked immunosorbent assays (IL‐6: R&D Systems, R600B, measured at 1:2 dilution; HA: Corgenix, 29001, measured at 1:10 dilution; Corgenix Medical Corp., Broomfield, CO, USA). Interferon‐γ (IFNγ), IL‐5, and IL‐8 concentrations were measured by rat‐specific 7‐plex multiplex immunoassay (Meso Scale Discovery, K15014C, measured neat; Meso Scale Diagnostics, Rockville, MD, USA). The remaining 7‐plex targets (IL‐1β, IL‐4, IL13, and TNFα) were below the level of detection for most samples. LPS was measured using the Endozyme kit from Biovendor (No. 609050) at a 1:1000 dilution with heating for 10 minutes at 70°. If a sample value was below the LLOD of minimal detectable difference (MDD), a value of one‐half the LLOD was imputed for the purpose of statistical analysis. The LLOD/MDD for the cytokines measured in this study were follows: IFNγ LLOD = 2.26 pg/mL, IL‐5 LLOD = 28.2 pg/mL, IL‐6 MDD = 21 pg/mL, IL‐8 LLOD = 0.68 pg/mL, and LPS LLOD = 0.005 EU/mL; HA LLOD is not reported. Average interassay CVs (%) were IFNγ = 4.0%, IL‐5 = 5.5%, IL‐6 = 7.4%, IL‐8 = 2.7%, HA = 5.9%, and LPS = 11.4%.

### Micro‐CT skeletal analysis

Immediately following death, right knee joints were harvested and frozen at −80°C in PBS‐wrapped gauze until μCT analysis. Thawed joints were then placed in fresh 4% paraformaldehyde at 4°C for 24 hours. We then removed most muscle and placed joints in fresh 4% paraformaldehyde in a specimen holder for scanning at room temperature. Images were acquired using a high‐resolution μCT system (vivaCT 40; Scanco Medical, Wangen‐Bruttisellen, Switzerland) at 15 μm voxel size, 55kVp, and 145 μA. Following scanning, the region of interest (ROI) was identified as ±15 slices from the midpoint of the tibial plateau along the anterioposterior axis. This 30‐slice ROI was manually segmented into medial and lateral proximal tibial subchondral bone and epiphysis trabecular bone. Global thresholding was used to segment calcified tissue from soft tissue, and linear attenuation of calcified tissue was scaled to bone density values using a hydroxyapatite calibration phantom. Morphometric parameters of fully calcified cortical and trabecular bone were determined using a direct three‐dimensional (3D) approach. Subchondral bone results were reported as bone mineral density (mg/cm^3^ of HA) and average thickness (mm). Trabecular bone results were reported as bone volume fraction (BV/TV), trabecular number (1/mm), trabecular thickness (mm), trabecular separation (mm), and bone mineral density.

### Histology and immunohistochemistry

Within 24 hours after μCT scanning, knees were placed in Cal‐Ex™ decalcifying solution (Fisher Scientific, No. CS510‐1D; Thermo Fisher Scientific, Hampton, NH, USA) and prepared for histological analysis as previously described for rats.^(^ ^39^ ^)^ Following decalcification and paraffin embedding, tissue blocks were sectioned in the coronal plane at 10‐μm thickness. Sections were then stained with hematoxylin, fast green, and Safranin O. Two experienced blinded graders evaluated cartilage degeneration using a semi‐quantitative histologic scoring system.^(^ ^39^ ^)^ Four sections throughout the medial and lateral femoral condyles and tibial plateau were scored. Scores from each site were presented as site‐specific averages and were also summed to generate a total joint summed score. Synovial hyperplasia and subsynovial inflammation were scored at a mid‐coronal section inferior to the lateral meniscus, as previously described.^(^ ^40^ ^)^ Osteophyte formation at the medial tibial plateau was scored as previously described.^(^ ^41^ ^)^ Leptin receptor immunostaining was performed with a rabbit anti‐Ob‐R polyclonal antibody (Santa Cruz, sc‐8325). Slides were deparaffinized, rehydrated, and incubated with antigen retrieval R‐Buffer A (EMS, 62706‐10) at 60°C for 18 hours. Slides were then treated with 2% H_2_O_2_ and blocked using 4% BSA and 10% donkey serum. Antibody was diluted 1:50 in 10% donkey serum and 0.1%Tween20 and applied to sections at 4°C overnight. Staining was detected using a Polink‐2 Plus HRP Rabbit with AEC chromogen kit (GBI Labs, D40‐18) following the manufacturer's instructions.

### Targeted cartilage proteomic analysis

Knee femoral and tibial articular cartilage was carefully dissected under a stereomicroscope from the contralateral limb not used for histology. Cartilage was immediately placed in TRIzol reagent (Ambion) on ice. Cartilage protein was isolated following manufacturer instructions and dissolved in 1% sodium dodecyl sulfate (SDS) for analysis. Protein was prepared and analyzed using selected reaction monitoring (SRM) mass spectrometry, as previously described.^(^ ^39^, ^42^ ^)^ Briefly, 8 pmoles of bovine BSA was added to each 20‐μg protein sample as an internal standard. Trypsin‐digested peptides were monitored on a TSQ Quantiva triple quadrupole mass spectrometry system (Thermo Fisher Scientific) linked to an Ultimate 3000 nanoflow HPLC system with 15 cm × 75 μm i.d. C18 reversed‐phase capillary column. 5‐μL aliquots were injected and the peptide eluted with a 60‐minute gradient of acetonitrile in 0.1% formic acid. The mass spectrometer operated in the SRM mode. Protein abundance was calculated as the geomean of two integrated peptide chromatographic peaks per protein and normalized to the sample total ion count and BSA internal standard. Targeted protein abundance was calculated for 93 proteins involved in cellular metabolism and redox homeostasis.

### Acute intra‐articular inflammatory challenge

We tested for a synergistic effect of leptin on joint inflammation by collecting synovial fluid at 6 and 18 hours following the intra‐articular injection of 1 μg IL‐1β (No. 201‐LB, R&D Systems; <1.0 EU endotoxin/μg protein) administered in 50 μL sterile saline (left knee) or saline alone (right knee). Immediately following euthanasia, synovial fluid was collected using the Whatman paper recovery method, as previously described.^(^ ^43^ ^)^ Synovial fluid recovery volume was estimated as the change in filter paper mass from before to after collection assuming a density of 1 μg/μL. Sample supernatants were frozen at −80°C until analysis. IL‐1β and IL‐8 concentrations were measured by rat‐specific 7‐plex multiplex immunoassay (K15014C, Meso Scale Discovery, measured neat). IL‐1β LLOD = 27.2 pg/mL (4.2% interassay CV) and IL‐8 LLOD = 0.68 pg/mL (2.7% interassay CV). The remaining 7‐plex targets (IFNγ, IL‐4, IL‐5, IL‐13, and TNFα) were below the level of detection for most samples. CCL2 (MCP‐1) was measured neat or at a 1:2 dilution by mouse/rat solid‐phase sandwich ELISA (MJE00, R&D Systems). The minimal detectable dose for CCL2 was <2 pg/mL, with an interassay %CV of 6.2%. Synovial hyperplasia and subsynovial inflammation were scored at a mid‐coronal section inferior to the lateral meniscus, as previously described.^(^ ^40^ ^)^

### Statistical analysis

Study sample sizes were based on power analyses for OA histopathology and subchondral bone μCT, our primary outcomes. Using data from our prior study of leptin mutant mice,^(^ ^29^ ^)^ *n* = 8 animals per group was estimated to provide 80% power to detect a 30% difference in mean OA scores (STDEV 24% of the mean) with a significance level of *p* = 0.05 (one‐tailed). This sample size was estimated to provide 99% power to detect a 30% difference in trabecular BV/TV (STDEV 15% of the mean), with a significance level of *p* = 0.05 (one‐tailed). One lean Zucker animal and one obese Zucker animal died prior to study completion (cause unknown). A reduced sample size to *n* = 7 per group lowered the a priori power estimate to 76%. Data were evaluated in Prism for macOS (version 9.4.1) for normality and homoscedasticity to determine the appropriate parametric or nonparametric statistical model and to perform data log transformation, if required. To minimize multiple statistical comparisons, we prioritized the use of two‐way ANOVA for comparisons involving two factors when the data set met the assumptions of this statistical model. When significant factor or interaction effects were observed, we performed post hoc comparisons to evaluate specific between‐group differences. The specific test for each comparison is summarized in Table S1. Data are shown as box and whisker plots with individual data points or as mean values ±95% confidence intervals (CIs).

## Results

### Development of obesity in pair‐fed obese Zucker rats

**Fig. 1:**
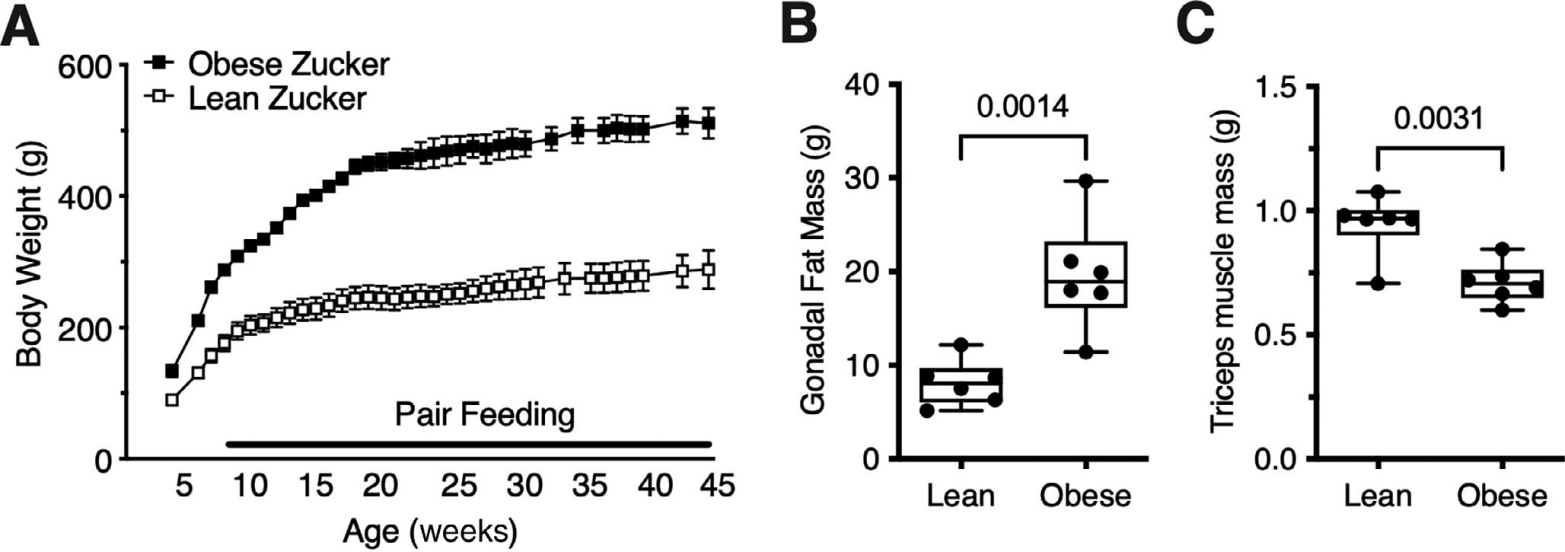
Body composition in lean and obese Zucker rats. (A) Obese Zucker rats were pair‐fed to age‐matched lean Zucker rats by gradually restricting the quantity of food provided to obese rats based on the average daily quantity of food consumed in the prior week by lean rats. Even with extended pair feeding, the body weight of obese Zucker rats was substantially greater than that of lean Zucker rats throughout the experiment's duration. Data points represent mean ± 95% CI (*n* = 7 per group). (B) Gonadal fat pad mass, which was significantly greater in obese Zucker rats versus lean Zucker rats, was measured by gross dissection following euthanasia. Individual animal data are shown as closed circles. Boxes represent 25th to 75th percentiles, horizontal line indicates median, and whiskers span minimum to maximum values. (C) Tricep muscle mass, which was significantly less in obese Zucker rats than in lean Zucker rats, was measured by gross dissection following euthanasia. Individual animal data are shown as closed circles. Boxes represent 25th to 75th percentiles, horizontal line indicates median, and whiskers span minimum to maximum values. Gonadal fat and muscle mass data were inadvertently not collected for the first lean and obese animals euthanized, reducing the sample size for these outcomes to *n* = 6 per group.

To control for hyperphagia that occurs in leptin receptor mutant obese Zucker rats, obese Zucker rat food availability was transitioned to the average daily quantity of food consumed in the prior week by age‐matched lean Zucker rats. During this pair‐feeding period, obese Zucker rats still gained about twice as much body weight compared to lean control rats (Figure 1A). At the end of the study, obese Zucker rats weighed 77% more than the control Zucker rats (*p* < 0.0001). The increased body weight was primarily due to an increase in adiposity. For example, gonadal fat pad mass was nearly twofold greater in the obese versus lean Zucker rats (Figure 1B), whereas tricep muscle mass was 25% less in obese versus lean Zucker rats (Figure 1D). Thus, even with matched food consumption, impaired leptin signaling significantly increased adiposity in female obese Zucker rats compared to lean controls.

### Obese Zucker rats developed focal OA lesions and osteophytes

**Fig. 2:**
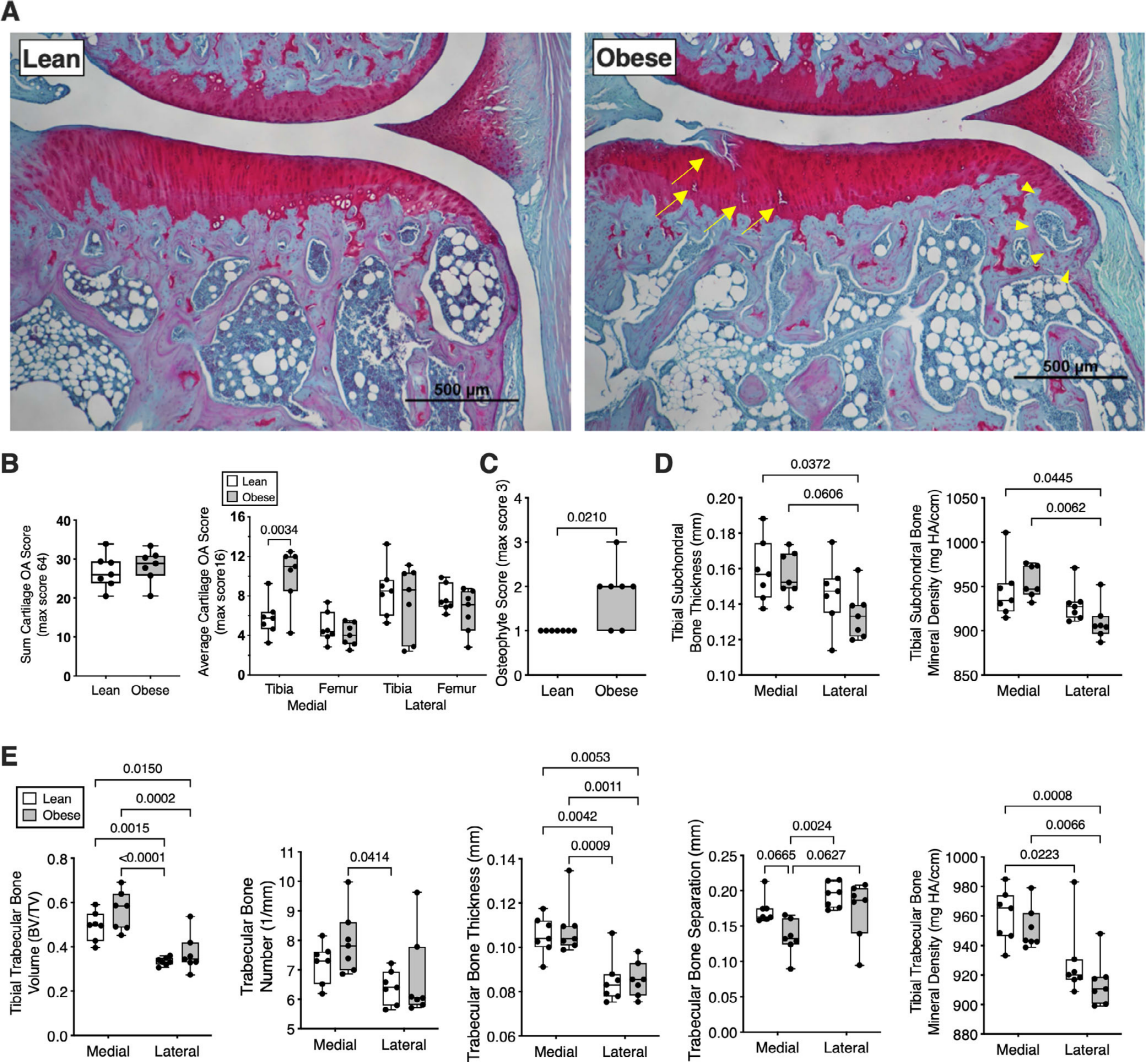
Knee joint cartilage and bone structure differences in lean versus obese Zucker rats. (A) Representative mid‐coronal medial tibial plateau histological comparison shows presence of focal cartilage lesions (yellow arrows) and osteophyte (yellow arrow heads) in an obese Zucker rat. Scale bar is 500 μm. (B) Cartilage OA pathology scores summed for all joint sites (left panel) or separated by joint site (right panel). Individual animal data are shown as closed circles (*n* = 7 per group). Boxes represent 25th to 75th percentiles, horizontal line indicates median, and whiskers span minimum to maximum values. (C) Osteophyte score comparison between lean and obese Zucker rats. Individual animal data are shown as closed circles. (D) μCT‐derived measurements of tibial subchondral bone thickness and bone mineral density. Open boxes = lean Zucker data, gray boxes = obese Zucker data. (E) Proximal tibial epiphysis trabecular bone morphometric data based on μCT analysis. Individual animal data are shown as closed circles. Boxes represent 25th to 75th percentiles, horizontal line indicates median, and whiskers span minimum to maximum values. Two‐way ANOVA *p* values provided in Table S1. Post hoc paired comparisons (*p* < 0.10) shown.

**Fig. 3:**
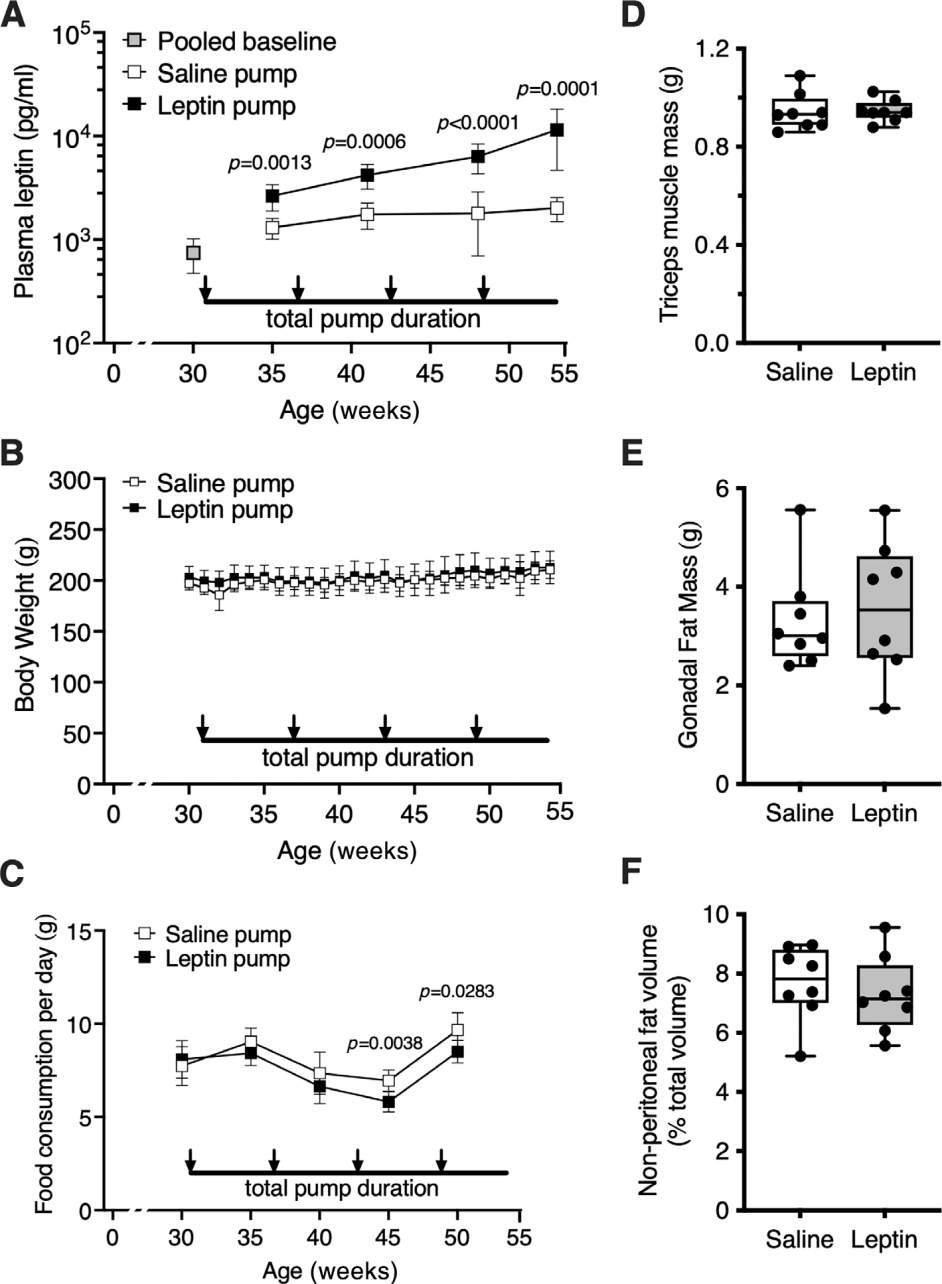
Physiologic response to leptin infusion in F344BN F1 hybrid rats. (A) Plasma leptin concentrations before (pooled baseline) or after insertion of mini‐osmotic pumps filled with 1 g/mL recombinant rat leptin (leptin pump) or saline (saline pump). Arrows show timing of four serial pump replacements over a 23‐week period (i.e., 35‐ to 42‐day intervals). Based on a diffusion rate of 0.15 μL/h, leptin pump rats received 3.6 μg leptin per day, which significantly increased plasma leptin over time. *P* values show post hoc age‐matched paired comparisons between leptin and saline pump groups. Data points represent mean ± 95% CI (*n* = 8 per group). (B) Weekly body weights did not differ between rats in saline and leptin pump groups. Data points represent mean ± 95% CI (*n* = 8 per group). (C) Average daily food consumption was modestly lower in leptin pump rats versus saline pump rats following third and fourth osmotic pump replacements. Data were collected over a 5‐day period to generate a daily average at indicated intervals. *P* values show post hoc age‐matched paired comparisons between leptin (*n* = 8) and saline (*n* = 8) pump groups. (D) Tricep muscle mass was measured by gross dissection following euthanasia and was not altered by leptin infusion. Individual animal data are shown as closed circles. Boxes represent 25th to 75th percentiles, horizontal line indicates median, and whiskers span minimum to maximum values. (E) Gonadal fat pad mass, which was measured by gross dissection following euthanasia, was also similar between leptin‐ and saline‐treated animals. (F) Nonperitoneal adipose tissue volume was quantified by MRI 16 weeks after initiation of leptin infusion. Data are reported as fraction of adipose to nonadipose tissue volume using “water‐suppressed” image sequences and automated segmentation procedures of user‐defined anatomic regions. No differences were observed between leptin‐ and saline‐treated groups.

We quantified cartilage OA pathology in Zucker rats at four knee joint sites: medial femur, medial tibia (Figure 2A), lateral femur, and lateral tibia. When scores were summed for all sites, there was no difference in cartilage OA pathology between lean and obese Zucker rats (Figure 2B). However, when genotypes were compared at each anatomic site, we observed a significant interaction between genotype and anatomic site due to a nearly twofold increase in cartilage OA pathology in obese Zucker rats at the medial tibial plateau (Figure 2B). The pathology was characterized as focal lesions that penetrated down to the tidemark (Figure 2A). In addition, obese Zucker rats developed greater osteophytes compared to lean animals (Figure 2A,C). μCT analysis revealed significant site‐specific differences, but no genotype‐dependent differences, in subchondral bone thickness or bone mineral density, with both values being greater in the medial versus lateral side (Figure 2D). Trabecular bone parameters were also mostly greater in the medial compared to the lateral compartment independent of genotype (Figure 3E). However, there was less trabecular bone separation in obese versus lean Zucker rats (*p* = 0.0088), which was associated with a trend for increased relative trabecular bone volume (BV/TV) (*p* = 0.0636) (Table S1). Synovial hyperplasia and inflammation were either absent or mild for both lean and obese Zucker rats (mean scores ≤1.0 for each parameter; statistical outcomes included in Table S1).

### Minimal effect of chronic leptin stimulation on systemic metabolic parameters

We tested the effect of chronically elevated systemic leptin on knee OA by treating lean female F344BN F1 hybrid rats with exogenous recombinant rat leptin for 23 weeks using osmotic pumps. Leptin infusion significantly increased plasma leptin concentration (*p* < 0.0001) in a time‐dependent manner (*p* = 0.001), resulting in 5.6‐fold greater plasma leptin concentrations at the completion of the study (Figure 3A). Despite increased circulating leptin, body weight was not altered (Figure 3B), although food consumption was modestly reduced after 15 weeks of treatment (*p* = 0.0426) (Figure 3C). We did not observe any significant effects of leptin infusion on tricep muscle mass (Figure 3D), gonadal fat mass (Figure 3E), or nonperitoneal fat volume normalized to total nonperitoneal volume (Figure 3F).

### Chronic leptin stimulation induced mild to moderate knee cartilage pathology and bone changes

**Fig. 4:**
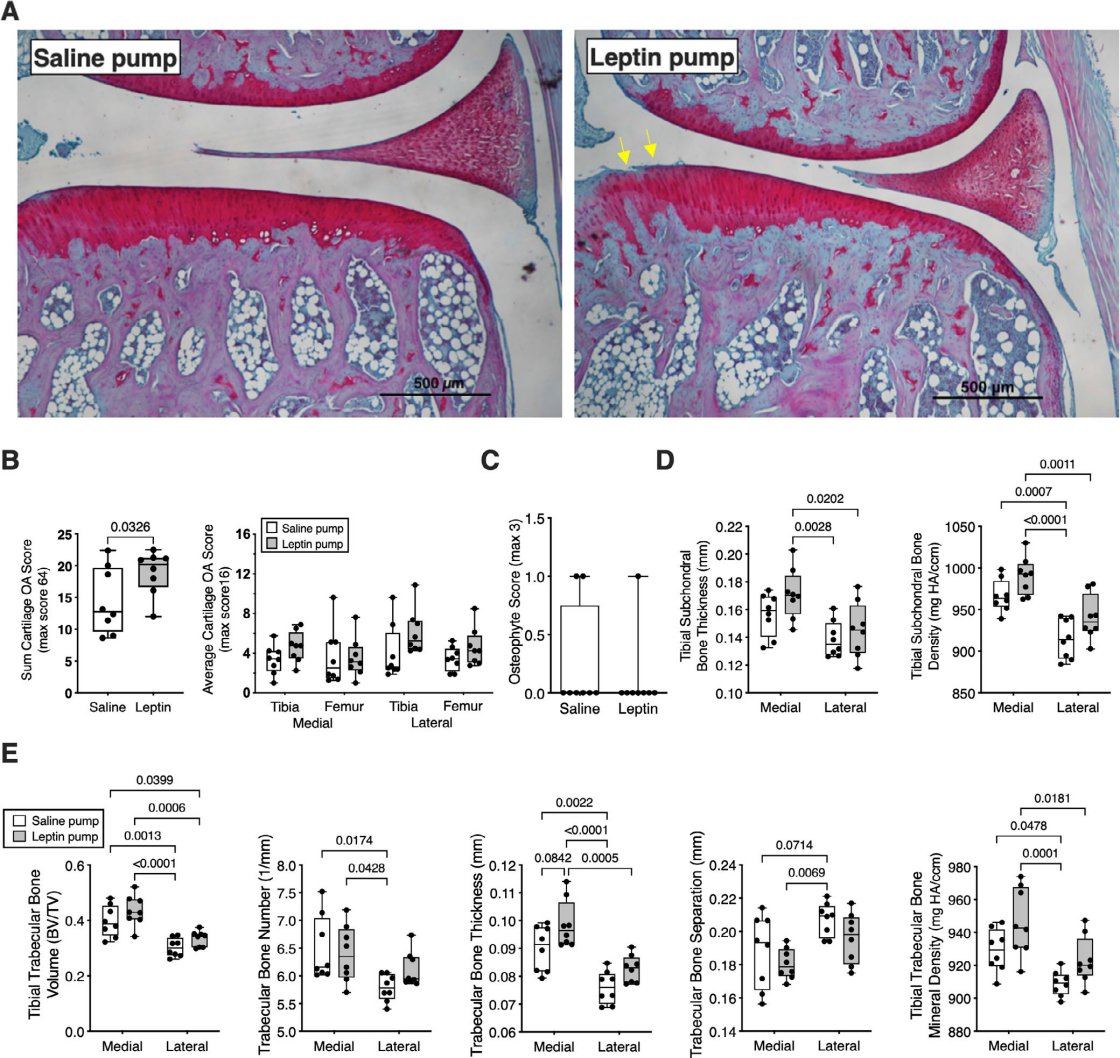
Knee joint cartilage and bone structure differences in leptin versus saline pump‐infused F344BN F1 hybrid rats. (A) Representative medial tibial plateau histological comparison shows presence of superficial cartilage fibrillations and reduced Safranin O staining (yellow arrows) in a leptin‐infused F344BN F1 hybrid rat. Scale bar is 500 μm. (B) Cartilage OA pathology scores summed for all joint sites (left panel) or separated by joint site (right panel). Individual animal data are shown as closed circles. Boxes represent 25th to 75th percentiles, horizontal line indicates median, and whiskers span minimum to maximum values. (C) Osteophyte score comparison between saline‐ and leptin‐treated rats shows minimal osteophyte development in either group. Individual animal data are shown as closed circles (*n* = 8 per group). (D) μCT‐derived measurements of tibial subchondral bone thickness and bone mineral density. Open boxes = saline group data, gray boxes = leptin group data. (E) Proximal tibial epiphysis trabecular bone morphometric data based on μCT analysis. Individual animal data are shown as closed circles. Boxes represent 25th to 75th percentiles, horizontal line indicates median, and whiskers span minimum to maximum values. Two‐way ANOVA *p* values provided in Table S1. Post hoc paired comparisons (*p* < 0.10) shown.

When scores were summed for all sites, we observed a 30% increase in cartilage OA pathology in rats receiving chronic leptin infusion (*p* = 0.0326), one‐tailed unpaired *t*‐test; mean difference (95% CI) = 4.48 (−0.26 to 9.22) (Figure 4A,B). Cartilage pathology was primarily characterized by surface fibrillation and reduced Safranin O staining across most anatomic sites (Figure 4A,B). Synovial hyperplasia and inflammation were either absent or mild (scores ≤1) for saline control animals, and leptin infusion did not alter this pattern (Table S1). Chronic leptin infusion also did not induce osteophyte formation (Figure 4A,C). μCT analysis showed significant site‐specific and leptin infusion effects on subchondral and trabecular bone features. As observed in Zucker rats, subchondral bone thickness and bone mineral density were both greater in the medial versus lateral side (Figure 4D). Leptin infusion significantly increased subchondral bone mineral density (*p* = 0.005) and showed a trend for increased subchondral bone thickness (*p* = 0.0806) (Figure 4D). Leptin infusion also increased trabecular bone mineral density (*p* = 0.0052) and altered several trabecular bone parameters, including a 10% increase in the relative trabecular bone volume (*p* = 0.0396) and trabecular bone thickness (*p* = 0.0043). Like Zucker rats, all the trabecular bone features, except for bone separation, were greater in the medial versus lateral compartment of F344BN F1 hybrid rats (Figure 4E) (Table S1).

**Fig. 5:**
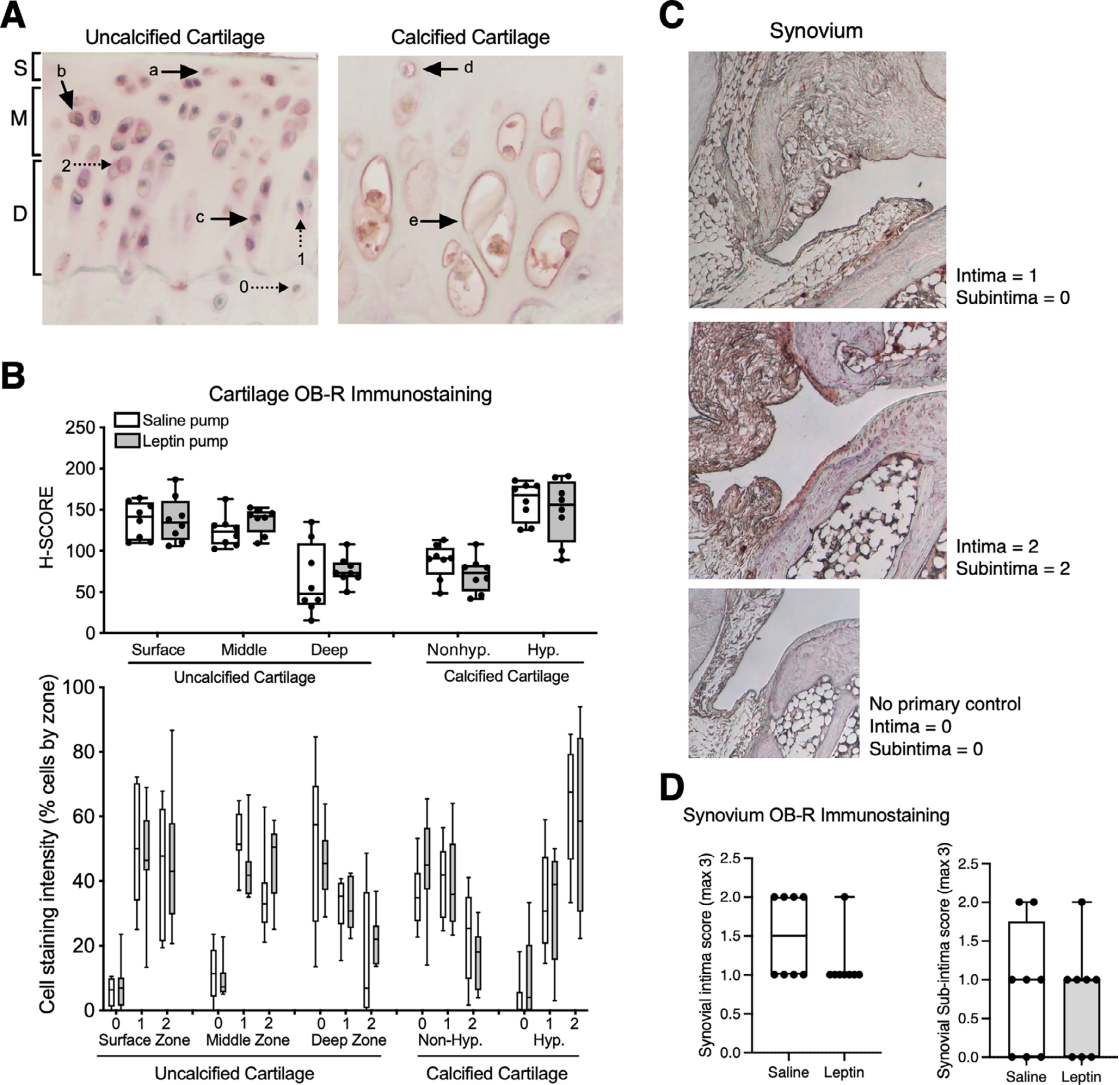
Chronic leptin treatment did not alter leptin receptor immunostaining in cartilage or synovium of F344BN F1 hybrid rats. (A) Representative immunohistochemical images of rat knee cartilage stained with leptin receptor (Ob‐R) antibody. Stained cells were counted in surface zone (a), middle zone (b), and deep zone (c) within uncalcified cartilage (left panel). Staining in nonhypertrophic (d) and hypertrophic (d) chondrocytes in calcified cartilage was also evaluated (right panel). Staining intensity was categorized as follows: 0 – no staining, 1 – weak staining, 2 – moderate to intense staining. (B) Chronic leptin infusion did not alter the H‐SCORE of leptin receptor staining across cartilage zones (upper panel), and it also did not alter the percentage of cells in each staining intensity category (lower panel); *n* = 8 per group. Data are presented as means ±95% CI. Two‐factor ANOVA revealed a significant effect of cartilage zone (*p* < 0.0001) but not leptin infusion on chondrocyte anti‐Ob‐R staining. (C) Representative anti‐Ob‐R staining images of synovium from mid‐coronal sections inferior to lateral meniscus. (D) Chronic leptin infusion did not alter anti‐Ob‐R staining in synovium (*n* = 8 per group).

Given that leptin and leptin receptor (Ob‐R) expression has been associated with OA lesions in human cartilage,^(^ ^18^, ^19^ ^)^ we investigated the effect of systemic leptin infusion on chondrocyte and synovium leptin receptor immunostaining. We used a semi‐quantitative H‐SCORE system to grade leptin receptor staining in different cartilage zones^(^ ^44^ ^)^ (Figure 5A). We found that chronic leptin infusion did not alter leptin receptor expression in any cartilage zone (Figure 5B). However, we observed site‐specific leptin receptor staining patterns. In uncalcified cartilage, leptin receptor staining was greater in the surface and middle zones compared to the deep zone. In calcified cartilage, leptin receptor staining was greater in hypertrophic chondrocytes compared to nonhypertrophic chondrocytes. Ob‐R staining was also evaluated in the synovial intima and subintima inferior to the lateral meniscus using a 0–3 scale based on increasing staining intensity and breadth (Figure 5C). Ob‐R staining was generally greater along the intima compared to the subintima, although there were no significant differences with leptin infusion compared to saline control (Figure 5D).

### Contrasting effects of body weight and leptin on metabolic and antioxidant protein content in cartilage

**Fig. 6:**
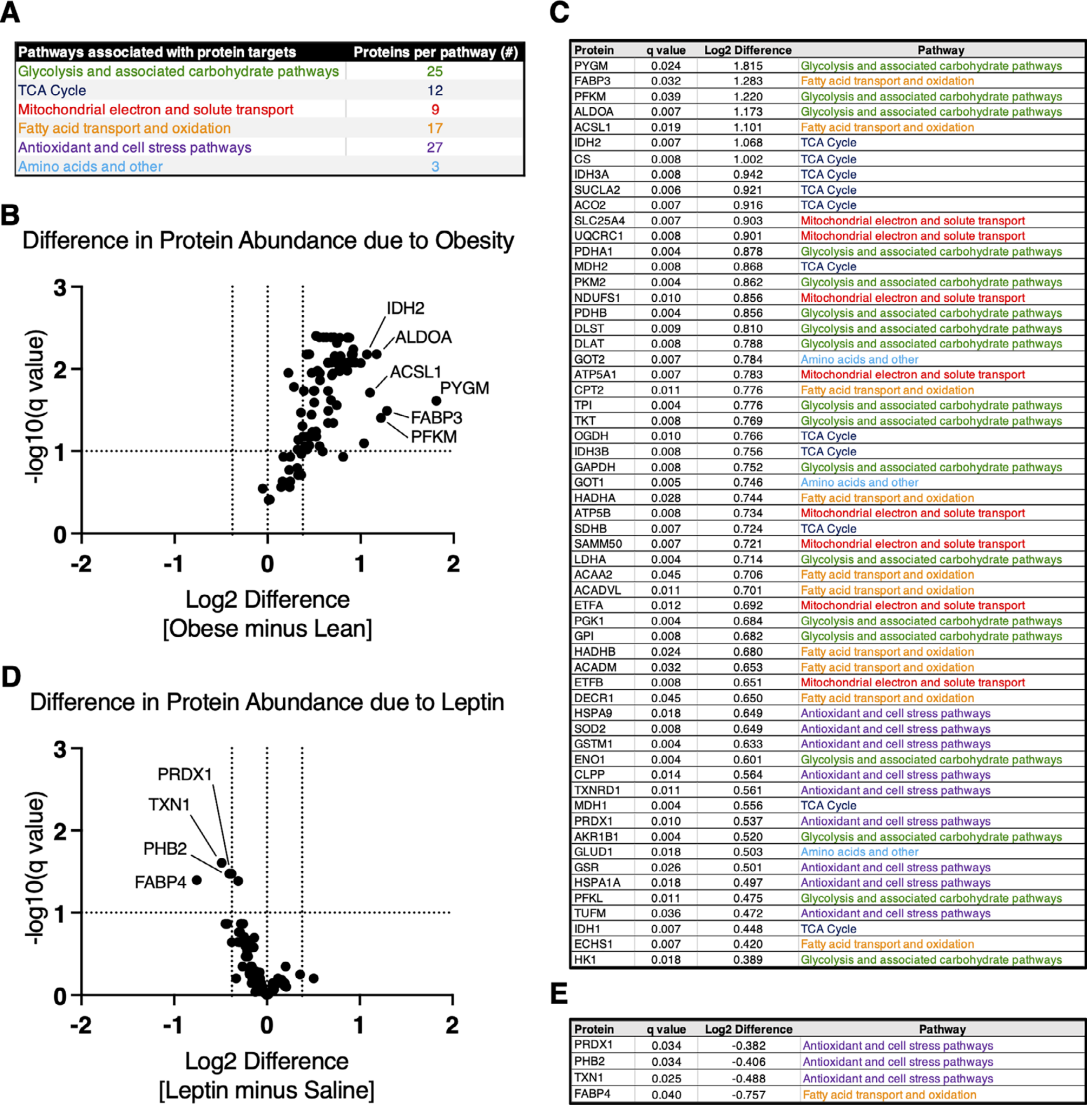
Targeted proteomic analysis reveals contrasting effects of body weight and leptin on metabolic and antioxidant protein content in cartilage. (A) We conducted a targeted quantitative analysis of cellular protein content to better understand the independent effects of body weight and leptin on cartilage. We evaluated >90 proteins in total (Table S2), which covered a range of metabolic, antioxidant, and cell stress pathways. (B) Effect of obesity was evaluated as mean differences in normalized cartilage protein content between obese Zucker (*n* = 7) minus lean Zucker (*n* = 7) samples, presented as volcano plot. Proteins that differed in abundance were identified based on log2 differences >∣0.3785∣and false discovery rate‐adjusted significance (*q* < 10%). (C) Nearly two‐thirds of all targeted proteins were more abundant in cartilage from obese versus lean Zucker rats, including proteins from each pathway. Differentially abundant proteins were ranked in descending order based on log2 difference values. (D) Effect of leptin was evaluated as mean differences in normalized cartilage protein content between leptin‐infused (*n* = 8) minus saline‐infused (*n* = 8) animal samples, presented as volcano plot. Proteins that differed in abundance were identified based on log2 differences >∣0.3785∣and false discovery rate‐adjusted significance (*q* < 10%). (E) Only four of the target proteins were differentially abundant following leptin treatment. All four proteins were less abundant with leptin treatment versus saline, and most contributed to antioxidant and cell stress processes. Differentially abundant proteins were ranked in descending order based on log2 difference values. A complete list of the mean difference values in protein content for all proteins is provided in Table S2.

We conducted a targeted quantitative analysis of metabolism‐associated cellular protein content in knee articular cartilage to better understand the independent effects of body weight and leptin on cartilage. As in previous studies,^(^ ^13^, ^39^, ^42^, ^45^ ^)^ we evaluated >90 proteins involved in carbohydrate metabolism, fatty acid metabolism, amino acid metabolism, the tricarboxylic acid cycle, and antioxidant and cellular stress pathways (Figure 6A). We compared the mean difference in normalized protein content between lean and obese Zucker rats or saline‐ and leptin‐infused F344BN F1 hybrid rats for each of the target proteins (Table S2). Values were then expressed as volcano plots to identify altered proteins based on log2 differences >∣0.3785∣and false discovery rate‐adjusted significance (*q* < 10%) (Figure 6B,D). These comparisons showed substantial differences in the effect of body weight versus leptin infusion on cartilage metabolic protein content. The majority of cellular metabolic proteins were more abundant in the cartilage of obese versus lean Zucker rats (Figure 6B,C). Proteins involved in glycolysis, fatty acid metabolism, and the tricarboxylic acid cycle (i.e., Pygm, Fabp3, Pfkm, Aldoa, Acsl1, Idh2, and Cs) were all more than twofold more abundant in the cartilage of obese Zucker rats compared to lean rats (Figure 6C). In contrast, leptin infusion only altered the content of four proteins (i.e., Prdx1, Phb2, Txn1, and Fabp4), and these proteins were less abundant in leptin‐infused versus saline control rats (Figure 6D,E). These four proteins are involved in antioxidant and cell stress processes and fatty acid transport (Figure 6E).

### Leptin and body weight caused distinct changes in systemic inflammatory biomarkers

Approximately half of the serum pro‐ and anti‐inflammatory biomarkers were not sufficiently detected to be included in the analysis (i.e., IL‐1β, IL‐4, IL13, and TNFα). Of those that were detected, IFNγ, IL‐5, and IL‐8 were not altered between lean and obese Zucker rats or saline‐ and leptin‐infused F344BN F1 hybrid rats (Table 1). Although leptin is a proinflammatory mediator, leptin infusion without obesity led to lower values of IL‐6 compared to saline controls (156 pg/mL versus 231 pg/mL, *p* = 0.025). IL‐6 was not different between lean and obese Zucker rats. In contrast, HA was altered in Zucker but not F344BN F1 rats. Serum HA was measured as a biomarker of joint inflammation, and it was approximately fivefold greater in obese versus lean Zucker rats (26.6 versus 5.1 ng/mL, *p* = 0.007). We also evaluated serum LPS as a systemic inflammatory biomarker related to gut dysbiosis and dysregulated LPS scavenging. Although serum LPS was not significantly altered in either rat strain, the values were greater in obese versus lean Zucker rats (*p* = 0.093) and in leptin versus saline infused F344BN rats (*p* = 0.188) (Table 1). The considerable within‐group variation in LPS values suggests that more animals would be required to conduct an adequately powered analysis.

**Table 1:** Serum biomarkers

	Zucker strain				F344 BN F1 hybrid strain			
	Lean	Obese			Saline	Leptin		
	Mean [± SD]	Mean [±SD]	Mean difference [95% CI]	*p* value	Mean [± SD]	Mean [± SD]	Mean difference [95% CI]	*p* value
**IFNγ**	2.87 [2.39]	1.13 [0.0]	−1.74 [−4.25 to 0.76]	0.1818	3.55 [2.36]	544.5 [1327]	541 [−852 to 1934]	0.9351
**IL‐5**	48.8 [26.9]	38.7 [19.5]	−10.1 [−40.7 to 12.5]	0.4746	60.9 [55.7]	193.9 [424.6]	133 [−312 to 578]	0.3723
**IL‐6**	147.0 [15.6]	136.1 [34.2]	−10.9 [−47.2 to 25.4]	0.4936	230.6 [65.7]	155.7 [18.1]	−74.8 [−144 to −6.05]	0.0254
**IL‐8**	37.0 [45.1]	80.4 [66.5]	43.3 [−31.1 to 118]	0.2158	12.9 [15.0]	36.9 [49.0]	24.0 [−27.4 to 75.4]	0.4459
**HA**	5.09 [3.92]	26.57 [20.20]	21.5 [0.3 to 42.6]	0.0067	7.51 [11.71]	2.75 [2.15]	−4.76 [−17.0 to 7.5]	0.4395
**LPS**	442 [790]	1865 [3007]	1423 [−1370 to 4216]	0.0927	24.4 [57.4]	37.0 [51.4]	12.6 [−45.9 to 71.0]	0.1879

*Note*: Serum and plasma samples were collected between 9:00 and 11:00 AM by cardiac puncture under anesthesia immediately prior to death. IFNγ, IL‐5, and IL‐8 (KC) were measured without dilution using MDS 7‐plex kit (Meso Scale Diagnostics) (K15014C) (undetected: IL‐1β, IL‐4, IL13, and TNFα). IL‐6 was measured at a 1:2 dilution by ELISA (R&D, R6000B), and HA was measured by ELISA at a 1:10 dilution (Corgenix, 29001). LPS was measured at a 1:1000 dilution using Biovendor's Endozyme kit. All units are pg/mL except for HA (ng/mL) and LPS (EU/mL). *n* = 6 per group. Statistical details are provided in Table S1.

### Systemic leptin infusion increased synovial fluid IL‐1β concentration in response to an acute intra‐articular challenge with IL‐1β

**Fig. 7:**
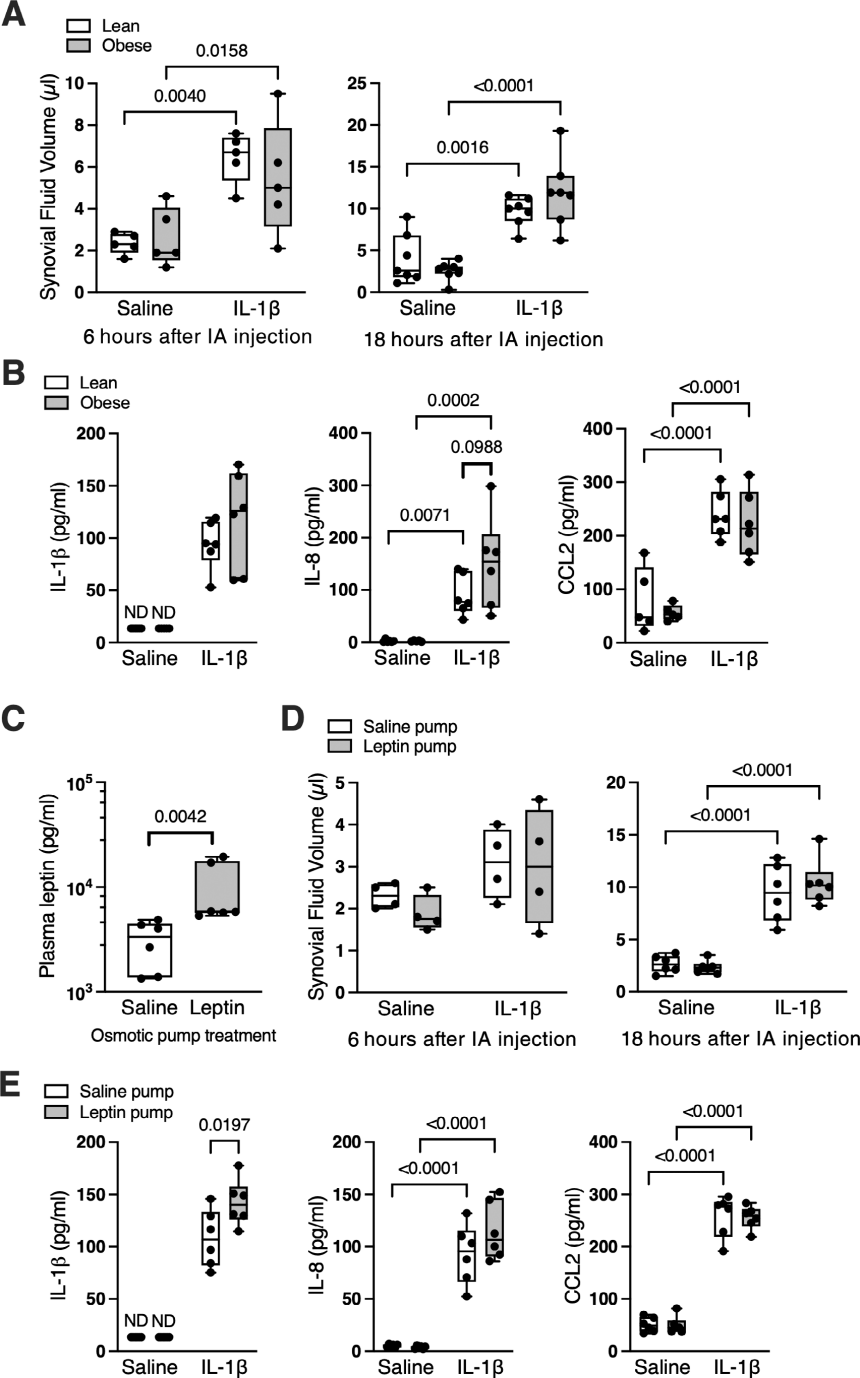
Systemic leptin infusion increases inflammation following acute intra‐articular challenge with IL‐1β. (A) Effect of intra‐articular injection of 1 μg IL‐1β on volume of synovial fluid recovered in 10‐ to 11‐week‐old lean and obese Zucker rats at 6 hours (left panel; *n* = 5 per group) or 18 hours (right panel; *n* = 7 per group) after injection. Synovial effusion increased with time following IL‐1β challenge, although no differences were observed between lean and obese Zucker rats. Individual animal data are shown as closed circles. Boxes represent 25th to 75th percentiles, horizontal line indicates median, and whiskers span minimum to maximum values. Two‐way ANOVA *p* values provided in Table S1. Post hoc paired comparisons (*p* < 0.10) shown. (B) Synovial fluid concentrations of IL‐1β, IL‐8, and CCL2 were increased at 18 hours following IL‐1β injection, but no differences were observed between lean and obese Zucker rats (*n* = 5–6 per group). (C) Plasma leptin concentration was increased in 11‐month‐old F344BN F1 hybrid rats after recombinant rat leptin infusion via an osmotic pump for 23 days compared to saline controls (*n* = 6 per group). (D) Effect of intra‐articular injection of 1 μg IL‐1β on volume of synovial fluid recovered in 12‐month‐old saline‐ versus leptin‐infused F344BN F1 hybrid rats at 6 hours (left panel; *n* = 4 per group) or 18 hours (right panel; *n* = 6 per group) after injection. No differences were observed between saline and leptin infused rats, and synovial effusion was only significantly elevated 18 hours following IL‐1β challenge. (E) Synovial fluid concentrations of IL‐1β, IL‐8, and CCL2 were increased at 18 hours following IL‐1β injection (*n* = 6 per group). Systemic leptin infusion further increased synovial fluid IL‐1β (left panel). ND = not detected.

**Fig. 8:**
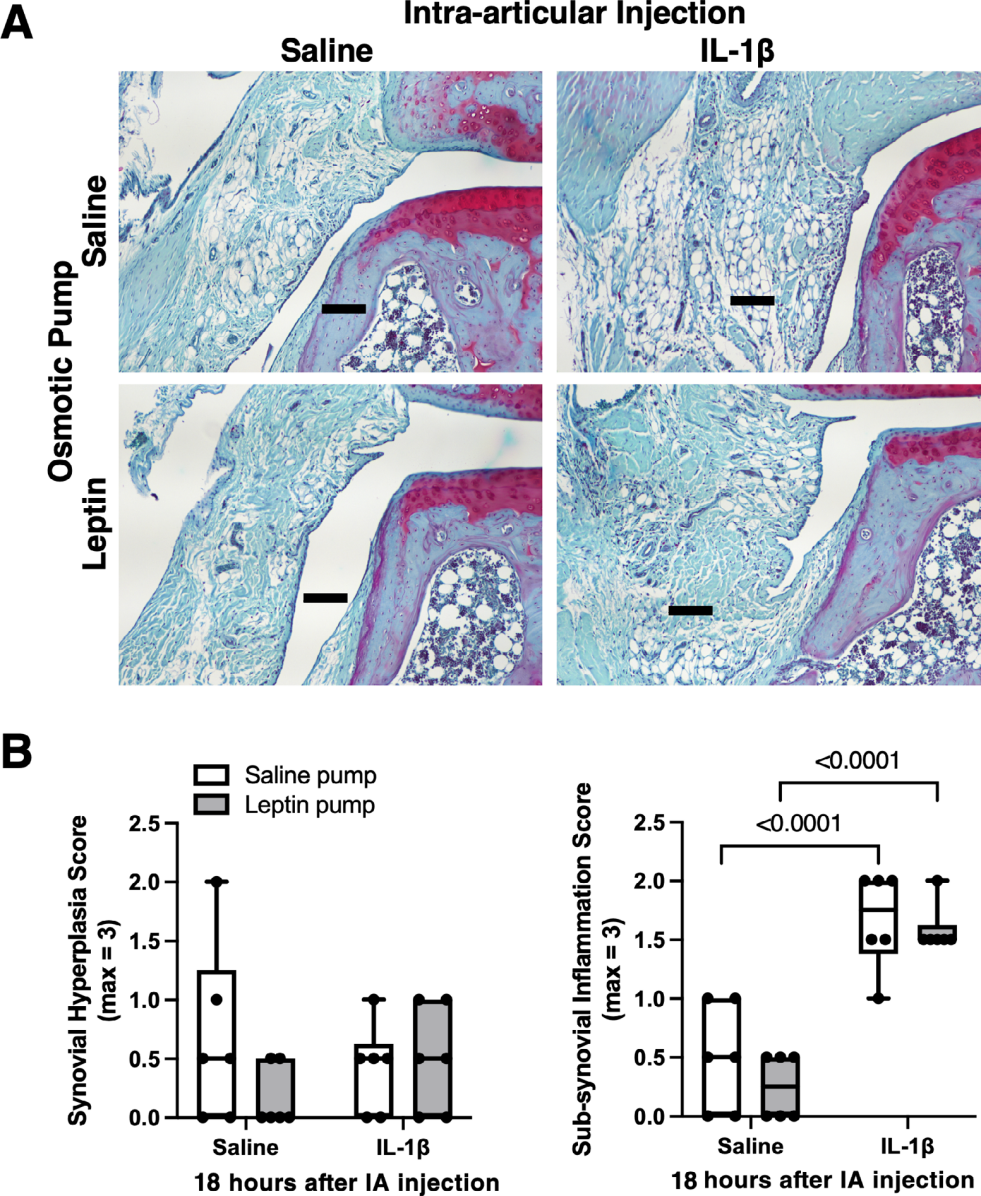
Effect of acute intra‐articular challenge with IL‐1β on histologic evidence of synovial inflammation. (A) Representative mid‐coronal sections inferior to lateral meniscus stained with hematoxylin, fast green, and Safranin O. Images are from saline and leptin pump‐treated animals 18 hours after intra‐articular injection with saline or IL‐1β. Scale bar is 100 μm. (B) Intra‐articular injection with IL‐1β increased subsynovial thickness and cellular infiltration (right panel) in both saline‐ and leptin‐infused F344BN F1 hybrid rats without altering synovial hyperplasia (left panel). Individual animal data are shown as closed circles (*n* = 6 per group). Boxes represent 25th to 75th percentiles, horizontal line indicates median, and whiskers span minimum to maximum values. Two‐way ANOVA *p* values provided in Table S1. Post hoc paired comparisons (*p* < 0.10) shown.

Based on previous in vitro reports showing that leptin synergistically increased cellular inflammation,^(^ ^21^ ^)^ we tested for a synergistic effect of leptin on synovial fluid inflammation in vivo following an acute intra‐articular injection of 1 μg IL‐1β. We also tested the responses in lean and obese Zucker rats to determine if obesity without leptin signaling modified the outcomes. Note that, unlike the prior comparisons, these outcomes were conducted in 11‐month‐old F344BN F1 hybrid rats receiving a much shorter (23‐day) leptin infusion or in young (10‐ to 11‐week‐old) lean and obese Zucker rats. Intra‐articular IL‐1β caused a similar degree of synovial effusion in lean and obese Zucker rats at both 6 hours (*p* = 0.0007) and 18 hours after stimulation (*p* < 0.0001) (Figure 7A). IL‐1β stimulation significantly increased the concentration of IL‐1β, IL‐8, and CCL2 in the synovial fluid to a similar extent in lean and obese Zucker rats, although there was a trend for greater IL‐8 in obese Zucker rats (*p* = 0.0988; Figure 7B). Even with the shorter duration of leptin infusion, plasma leptin was significantly elevated versus saline control (*p* = 0.0042; Figure 7C). Intra‐articular IL‐1β stimulation caused mild synovial effusion after 6 hours (*p* = 0.0621) and significant effusion at 18 hours (*p* < 0.0001) (Figure 7D). Systemic leptin infusion significantly elevated synovial fluid IL‐1β following intra‐articular IL‐1β stimulation compared to saline infusion controls (*p* = 0.0197; Figure 7E). Intra‐articular IL‐1β also increased synovial fluid IL‐8 and CCL2, although to a similar extent in leptin and saline infusion groups (Figure 7E). Histological analysis showed that intra‐articular IL‐1β increased subsynovial inflammation without altering synovial hyperplasia in F344BN F1 rats, although there were no differences in either parameter between leptin‐ and saline pump‐infused animals (Figure 8A,B).

## Discussion

The obesity‐associated adipokine leptin has been of interest to the OA research community for nearly two decades as a soluble proinflammatory mediator linking obesity and OA.^(^ ^18^, ^27^, ^28^ ^)^ The goal of this study was to examine the role of leptin in OA pathogenesis using preclinical animal models selected to experimentally manipulate either leptin or body weight. We hypothesized that leptin was necessary to induce OA in obese rats but not sufficient by itself to induce OA in lean rats lacking systemic metabolic inflammation associated with obesity. However, our results did not support this hypothesis. First, we showed that leptin signaling was not necessary to induce OA in obese rats based on our observation that leptin receptor mutant obese Zucker rats developed focal cartilage lesions and osteophytes in the medial tibial compartment. Second, we showed that chronic leptin infusion caused mild superficial fibrillation and reduced Safranin O staining of knee articular cartilage and subchondral bone sclerosis without altering body weight compared to saline infusion. Thus, these findings support the conclusion that both leptin‐dependent and independent factors link obesity and OA.

As with previous studies,^(^ ^26^ ^)^ our results suggest that one way leptin promotes OA is by modulating inflammation. Many prior OA‐related studies examined the proinflammatory role of leptin in vitro or following acute in vivo leptin administration, finding that leptin increased inflammation in a synergistic manner under inflammatory conditions.^(^ ^21^, ^26^ ^)^ We found that 3 weeks of exogenous systemic leptin infusion increased synovial fluid IL‐1β concentration 18 hours after intra‐articular administration of IL‐1β. However, without IL‐1β stimulation, exogenous leptin infusion was not sufficient to increase the concentration of synovial fluid IL‐1β, IL‐8, or CCL2. These findings are consistent with leptin functioning as a synergistic mediator of joint inflammation. We note, though, that impaired leptin signaling did not inhibit the induction of these proinflammatory molecules by IL‐1β in obese versus lean Zucker rats. Obese Zucker rats develop metabolic inflammation at a young age,^(^ ^46^ ^)^ which may offset a protective effect of disrupted leptin signaling. While we do not know which cell types mediate these findings, we hypothesize that infiltrating myeloid cells are responsible for the leptin‐dependent increase in IL‐1β. Monocytes and macrophages are sensitive to leptin‐dependent activation^(^ ^47^ ^)^ and are primary intra‐articular sources of IL‐1β.^(^ ^48^ ^)^ Myeloid cells were likely recruited to the joint because monocyte chemotactic protein CCL2 was significantly elevated following IL‐1β injection, and a histologic analysis confirmed the increase in subsynovial cellular infiltration.

Future studies are needed to dissect the relative contribution of systemic versus local leptin on joint inflammation following an injury. One potential leptin‐dependent mechanism is through high‐density lipoprotein (HDL)‐mediated endotoxin scavenging and chondrocyte cholesterol efflux.^(^ ^28^ ^)^ Unfortunately, we did not measure serum HDL, and this study was underpowered to evaluate group differences in serum LPS given the variability in these measurements, although there was a trend for increased LPS in obese versus lean Zucker rats. Changes in local leptin production from intra‐articular fat are also important to consider. We previously showed that ex vivo IL‐1β treatment of infrapatellar fat pads harvested from F344BN F1 rats reduced the secretion of leptin,^(^ ^49^ ^)^ suggesting that the synergistic proinflammatory effects of leptin may be held in check by a decrease in local leptin production after joint injury. Together, these data suggest that the relationship between leptin and inflammation is complex and context dependent.

Our findings also indicate a complex effect of sustained exogenous leptin infusion in the absence of obesity on systemic inflammation. Twenty‐three weeks of leptin infusion significantly reduced serum IL‐6. In addition, obesity in the absence of leptin signaling (i.e., obese Zucker rats) did not cause increased serum IL‐6. These findings are relevant to OA because IL‐6, like leptin, is associated with obesity, incident radiographic knee OA,^(^ ^50^ ^)^ and postinjury cartilage catabolism.^(^ ^51^ ^)^ The relationship between IL‐6 and OA, however, is not simple. IL‐6 is a pleiotropic cytokine that exerts context‐dependent proinflammatory and anti‐inflammatory actions.^(^ ^52^, ^53^ ^)^ For example, IL‐6 is proinflammatory when it is elevated in the plasma in individuals with obesity and metabolic syndrome. In contrast, IL‐6 is anti‐inflammatory when muscles transiently increase systemic IL‐6 levels following exercise. Genetic deletion of IL‐6 in mice causes adult onset of obesity,^(^ ^54^ ^)^ impaired insulin signaling,^(^ ^54^ ^)^ and the development of age‐associated knee OA in male animals.^(^ ^55^ ^)^ We do not know how chronic exogenous leptin administration reduces serum IL‐6, although a similar finding was previously reported for leptin‐deficient low‐density lipoprotein receptor knockout female mice administered leptin for 12 weeks.^(^ ^56^ ^)^ Leptin and IL‐6 share several mechanisms of regulation relevant to OA. Leptin and IL‐6 both signal through the JAK‐STAT3 pathway, which is negatively regulated by suppressor of cytokine signaling 3 (SOCS‐3).^(^ ^57^ ^)^ SOCS‐3 was previously shown to suppress the procatabolic effects of leptin in chondrocytes,^(^ ^25^ ^)^ and SOCS‐3 gene expression in cartilage is negatively associated with synovial fluid IL‐6 and MMPs 1 and 3 from patients with obesity undergoing knee replacement surgery.^(^ ^25^ ^)^ More work is needed to further examine how leptin and IL‐6 interact to modify OA pathogenesis, both systemically and in joint tissues.

Recent studies detail how leptin signaling, in addition to regulating inflammation, induces numerous changes in chondrocyte anabolic activity, protein homeostasis processes, and cell viability.^(^ ^26^ ^)^ Chronic leptin stimulation appears to have a biphasic effect on chondrocytes characterized by short‐term activation of the mTOR pathway and proanabolic processes that over time transition to favor catabolic processes and reductions in cell viability.^(^ ^26^ ^)^ Our proteomic analysis of cartilage suggests that a sustained moderate increase in systemic leptin, in the absence of obesity, has a relatively modest impact on articular chondrocyte metabolism and related processes. Those proteins that were altered were all reduced compared to control animals. Fatty acid binding protein 4 (FABP4) content was 40% lower in cartilage from leptin‐infused rats. FABP4 is a cytosolic fatty acid transport protein involved in lipid signaling, fatty acid efflux, and hormone‐like metabolic regulation,^(^ ^58^ ^)^ and there are recent reports of its potential role in OA pathogenesis.^(^ ^59^ ^)^ Additional downregulated proteins include thioredoxin1 (TXN1), prohibitin 2 (PHB2), and peroxiredoxin 1 (PRDX1). These proteins, along with several additional downregulated proteins that did not pass the multiple comparison‐adjusted statistical threshold (Table S2), are involved in cellular quality control and antioxidant processes. Consequently, a reduction in the content of these proteins may limit the ability of chondrocytes to adequately respond to cellular stress. Although metabolic activity may have been more greatly affected at an earlier period of leptin infusion, we were surprised that more metabolic and cell stress response proteins were not significantly altered after 23 weeks of elevated systemic leptin.

In contrast, many proteins were upregulated in cartilage from obese Zucker rats compared to lean Zucker rats, indicating a major effect of obesity on chondrocyte metabolism. Nearly two‐thirds of all targeted proteins representing all covered pathways were more abundant in cartilage from obese Zucker rats, and no proteins were less abundant in obese versus lean animals. Proteins involved in the tricarboxylic acid cycle, glycolysis, and associated carbohydrate metabolism processes were most enriched, followed by proteins involved in fatty acid transport and oxidation, antioxidants, and cell stress response pathways. These findings are distinct from our prior targeted proteomic studies comparing cartilage from control‐diet and high‐fat‐diet mice. Diet‐induced obesity upregulates proteins involved in fatty acid transport and oxidation and amino acid degradation without changes in glycolysis and associated carbohydrate metabolism proteins.^(^ ^13^, ^45^ ^)^ In the current study, lean and obese Zucker rats were fed the same 10% kcal fat diet, and obese Zucker rats were pair fed based on the consumption patterns of lean animals. Nevertheless, obese Zucker rats still gained significantly more weight and adipose tissue compared to lean animals due to impaired leptin signaling. These findings raise intriguing questions about the role of high dietary fat and/or leptin signaling in promoting a metabolic shift in chondrocytes favoring increased fatty acid oxidation. However, there are two important limitations for interpreting these data. First, the proteomic results are based on articular cartilage harvested from all knee sites, although obese Zucker rats developed increased OA in the medial tibial plateau. Therefore, these findings may reflect the effects of both OA and obesity. Second, protein was isolated from total cartilage homogenate that included intra‐cellular and extracellular proteins, such as collagens and proteoglycans. To account for variation in the total amount of protein that was available for analysis, we normalized results to total ion count as well as exogenous BSA to account for variation in sample processing. If changes in cellularity are not proportional to total ion counts, the findings may reflect an increase cell density in obese versus lean Zucker rat cartilage.

The current finding that leptin receptor mutant obese Zucker rats develop OA compared to nonmutant lean Zucker rats differs from our prior study involving leptin receptor mutant *db/db* mice, which did not develop OA despite extreme obesity.^(^ ^29^ ^)^ Several factors may contribute to these divergent findings. For example, the severity of obesity is greater in *db/db* mice versus obese Zucker rats based on the relative increase in body weight and visceral adiposity compared to each species' nonmutant control. A consequence of the extreme obesity in *db/db* mice is that the animals support much of their body weight on their abdomen and have low levels of cage activity. We unfortunately do not have direct measurements of either limb loading or cage activity for either species, but anecdotally there appears to be relatively less limb loading in *db/db* mice compared to obese Zucker rats. Another difference between the two studies is the diet composition and availability. In the *db/db* mouse study, animals were allowed ad libitum access to standard rodent chow. However, to better control for comparisons to high‐fat‐diet studies that use defined diets with lard as a fat source, we fed Zucker rats a defined 10% low‐fat diet that used lard as the fat source (D12450Bi, Research Diets). Although this quantity of fat is similar to that of rodent chow, chow does not include animal fat. Lard is primarily composed of saturated fat, which promotes inflammation and chondrocyte stress.^(^ ^12^, ^60^ ^)^ In addition, our current study involved pair feeding obese Zucker based on the average quantity of ad libitum food consumed by lean Zucker animals during the prior week. Although limiting the amount and duration of food availability is often associated with improved metabolic outcomes, we fed obese Zucker rats once during the natural resting period to blunt these metabolic benefits.^(^ ^61^ ^)^ Whether or not this disruption in peripheral circadian patterns contributed to the observed pathology is not known. However, based on the site‐specific and focal nature of the cartilage lesions extending to the tidemark, we suspect that the OA pathology observed in obese Zucker rats may be due to altered biomechanical stress in the knee joint.

While the goal of this study was to examine the role of leptin in OA pathogenesis using preclinical animal models selected to experimentally manipulate either leptin or body weight, it is important to recognize some important differences in these models. The lean versus obese Zucker rat comparison is based on a spontaneous heritable genetic mutation, which is present throughout life and causes a robust obesity phenotype with relatively minimal variation. In contrast, the leptin treatment model involves administration of exogenous recombinant leptin via serially implanted osmotic pumps. This process is susceptible to variation in mean leptin exposure due to time‐dependent changes in pump performance, individual animal clearance, and endogenous leptin production. The relatively lower summed OA scores and greater variance observed in F344BN F1 rats compared to Zucker rats may reflect these experimental differences. In addition, the mild increase in cartilage surface fibrillation and reduced Safranin O staining observed at most joint sites in F344BH F1 hybrid rats receiving systemic leptin treatment is consistent with the effects of a soluble factor that impacts all joint regions. Repeating the leptin treatment model in combination with an injury may further reveal important synergistic effects of leptin, inflammation, and biomechanical stress, as suggested in a recent study.^(^ ^15^ ^)^

In conclusion, we investigated the role of leptin in OA pathogenesis using preclinical animal models selected to experimentally manipulate either leptin or body weight. We believe that such studies are important because numerous OA risk factors (e.g., altered joint biomechanics, impaired tissue structure, and metabolic inflammation) covary with one another and with obesity itself,^(^ ^3^ ^)^ preventing a clear understanding of the etiology of obesity‐induced OA. Both exogenous systemic leptin infusion in the absence of obesity and obesity in leptin receptor mutant animals caused distinct knee OA phenotypes compared to the respective control animals. These data support the conclusion that obesity increases OA pathology through leptin‐dependent and independent pathways involving changes in systemic inflammation and cartilage homeostasis.

## Author Contributions

**Yao Fu:** Formal analysis; investigation; methodology; writing – original draft; writing – review and editing. **Albert Batushansky:** Data curation; formal analysis; software; visualization; writing – review and editing. **Michael Kinter:** Formal analysis; investigation; methodology; writing – review and editing. **Janet Huebner:** Formal analysis; methodology; writing – review and editing. **Virginia Kraus:** Methodology; writing – review and editing. **Timothy Griffin:** Conceptualization; data curation; formal analysis; funding acquisition; investigation; project administration; supervision; visualization; writing – original draft; writing – review and editing.

## Funding Information

Supported by an Arthritis Foundation Innovative Research Grant (Award 5344) and the National Institutes of Health (R01AG049058, P30GM114731, P30AG028716, P20GM103447). The content is the sole responsibility of the authors and does not necessarily represent the official views of the funding sources, which played no role in the conduct, writing, or submission of the manuscript for publication.

## Conflict of Interest

The authors have no conflicts of interest to declare.

### Peer Review

The peer review history for this article is available at https://www.webofscience.com/api/gateway/wos/peer-review/10.1002/jbm4.10754.

## Supporting information


**Appendix S1.** Supplementary Information

## Data Availability

Data that support the findings of this study are available from the corresponding author upon reasonable request.
