# Supplementary material for: Effects of Leptin and Body Weight on Inflammation and Knee Osteoarthritis Phenotypes in Female Rats
Source: JBMR Plus. 2023 May 5;7(7):e10754. doi: 10.1002/jbm4.10754 (PMC10339097; doi:10.1002/jbm4.10754)
Supplement: Supplementary file 1 — Appendix S1. Supplementary Information [file JBM4-7-e10754-s001.docx]

**Supplemental Materials**

**Effects of Leptin and Body Weight on Knee Osteoarthritis**

**Phenotypes in Female Rats**

**Contents:**

**Table S1: Statistics Summary**

**Table S2: Targeted Proteomic Analysis Results.** Note: Bold numbers indicate differences in protein content due to obesity (obese versus lean Zucker samples) or leptin (leptin pump versus saline pump F344BN samples) based on 95% confidence intervals of mean difference values that do not span zero. See Figure 6 in the main text for false discovery rate adjusted statistical analysis results.
